# Volatile fatty acid production from mesophilic acidogenic fermentation of organic fraction of municipal solid waste and food waste under acidic and alkaline pH

**DOI:** 10.1007/s11356-019-05394-6

**Published:** 2019-05-20

**Authors:** Yen-Keong Cheah, Carme Vidal-Antich, Joan Dosta, Joan Mata-Álvarez

**Affiliations:** 1 Department of Chemical Engineering and Analytical Chemistry, University of Barcelona, 08028 Barcelona, Catalonia Spain; 2 Water Research Institute, University of Barcelona, 08001 Barcelona, Catalonia Spain

**Keywords:** Mechanical-biological treatment plant, OFMSW, PHA, Mesophilic temperature, Mixed microbial cultures, VFA distribution

## Abstract

This study is focused on the effects of pH on the production of volatile fatty acids (VFAs) and their distribution through the acidogenic fermentation of source-sorted organic fraction of municipal solid waste (OFMSW) from a mechanical-biological treatment (MBT) plant, and food waste (FW) from a university canteen. In semi-continuous lab-scale digesters using OFMSW at a hydraulic retention time (HRT) of 3.5 days under acidic conditions (pH 6.0), the VFA concentration in the effluent increased to 9.8–11.5 g L^−1^ (VS content of the feedstock between 4.2 and 5.2% *w*/*w*), while its individual VFA profiling was similar to the influent which was already pre-fermented (namely, C_2_ 35–41%, C_3_ 18–22%, C_4_ 17–21%, and C_5_ 9–12%). When working with the same conditions but using FW as feedstock, an effluent with a VFA concentration up to 11.5 g VFA L^−1^ (FW with a VS content of 5.5% *w*/*w*) and a stable distribution of C_2_ and C_4_ acids (up to 60.3% and 12.9%, respectively) but with very low quantities of C_3_ and C_5_ acids (lower than 1.8 and 2.7%, respectively) was obtained. Anaerobic batch tests using FW revealed that alkaline pH near 9 could lead to higher VFA production with high acetic acid content when compared to pH 6. In the semi-continuous fermenters working at alkaline conditions (pH 9.5–10) using OFMSW and FW, an enhanced solubilization of organic matter was registered with respect to the fermenters working under acidic conditions. This fact was not reflected in a higher VFA production when using OFMSW as feedstock, probably due to free ammonia inhibition, since OFMSW was mixed in the MBT plant with supernatant from anaerobic digestion of this biowaste. However, when using FW, alkaline conditions lead to an enhanced VFA production with respect to the reactor working under acidic conditions, being acetic acid the predominant product, which represented up to 91% of the VFA spectrum obtained.

**Electronic supplementary material:**

The online version of this article (10.1007/s11356-019-05394-6) contains supplementary material, which is available to authorized users.

## Introduction

The rapid growth of human population and the global economy has led to a large amount of urban organic waste generation resulting in vital environmental problems in the world. These organic wastes include food waste (FW), which is the majority proportion, paper waste, small-sized plant waste, compostable materials, and other materials which are made from the mentioned resources. According to Waste Framework Directive (2008/98/EU), organic fraction of municipal solid waste (OFMSW) and other easily biodegradable solid substrates of urban origin have to be conveniently treated to reduce their impact and to recover energy and materials while disposal treatments (e.g., landfill or incineration) should be avoided. Landfilling has received criticizes due to greenhouse gas emission. Food Agriculture Organization in 2011 claimed that FW had potential to emit more than 3.1 billion tons of equivalent CO_2_ and if FW was a country, it would be the third biggest CO_2_ producer in the world. Considering the strong EU commitment toward the implementation of European circular economy, OFMSW is an ideal feedstock for biorefinery processes (Escamilla-Alvarado et al. 2017) since it is composed by carbohydrates (simple sugars and polysaccharides), proteins, and lipids that could be fermented without much difficulty. In comparison with OFMSW, total chemical oxygen demand (COD) of FW (90–170 g L^−1^) is usually almost half than that of OFMSW (150–350 g L^−1^) (Elbeshbishy et al. 2011; Lee et al. 2014; Strazzera et al. 2018). In any case, these two solid wastes are interesting substrates, as they are constantly generated with substantial amount, and, regardless of its possible heterogeneity, they could be efficiently treated through acidogenic fermentation using mixed microbial cultures to produce valuable products like volatile fatty acids (VFAs) and other short-chain organic compounds such as alcohols or lactic acid (Tang et al. 2018, 2019).

The acidogenic fermentation is a process which is based on hydrolysis and acidogenic phases from anaerobic digestion (AD) process. The hydrolysis occurs when complex organic matter (such as proteins, carbohydrates, and fats or oils) are broken down into simpler organic monomers (sugars, amino acids, and fatty acids) to be readily available for other bacteria. In acidogenic fermentation of complex biodegradable substrates, such as OFMSW and FW, the hydrolysis has been identified as a rate-limiting step, which is calling for optimizations of operating parameters during the process (Lim et al. 2008; Lee et al. 2014). In the acidogenic phase, the hydrolyzed organic monomers are consumed by groups of bacteria to produce VFA. These VFAs have several applications such as carbon source for biological nutrient removal (BNR) from municipal wastewater (Fang and Liu 2018), bioenergy with H_2_ (Slezak et al. 2017), biogas production (Mu et al. 2017), and biopolymer production (Valentino et al. 2015; Korkakaki et al. 2016). Polyhydroxyalkanoates (PHAs), for example, are a new generation of biopolymers which can be fully biodegraded, thus reducing its environmental impact. These PHA polymers are an interesting alternative to replace petrochemical derivative plastics which has raised awareness of its disposal problem and several pollution concerns, since PHA polymers and current commercialized plastics share similar thermoplastic properties (Morgan-Sagastume et al. 2010). As a future prospect, disposal of plastics waste can be minimized when innovative and environmentally safe biodegradable polymers are used in applications like packaging, agriculture, and health industry (Ahmed et al. 2018). In fact, VFAs are considered the most suitable substrates for PHA storage (Cai et al. 2009), and consequently high yield of VFA will increase the competence of bioplastics in the current plastics market.

Many researches on production of VFA have been carried in the last decades, focusing on fermentation strategies, process configuration, metabolic pathway analysis, and microbial characterization (Dias et al. 2006; Akaraonye et al. 2010; Korkakaki et al. 2016). Specific studies on process operational parameters, i.e., pH, temperature, hydraulic retention time (HRT), and organic loading rate (OLR), have been carried out (Jiang et al. 2013; Hao and Wang 2015; Wang et al. 2016) because of remarkable influences on generation of desired products caused by changing these process variables. Based on previous findings, these parameters lead to different metabolic pathways to produce a certain number of carbon-chain fatty acids. During the acidogenic fermentation process, pH can affect not only hydrolysis, but also acidogenesis (Neyens et al. 2004; Jiang et al. 2013) and can highly influence the VFA yield and distribution (Dareioti et al. 2014). Therefore, the optimal pH for acidogenic fermentation will favor these two steps to promote VFA production. Moreover, pH is important to avoid methanogenic bacteria activities, for example, operating the reactor out of the optimal pH range of 7.0–8.2 for methane production (Angelidaki and Sanders 2004; Chaganti et al. 2011). Also, several researches have proved that methanogenesis can be inhibited by increasing or decreasing to an extreme pH (Yuan et al. 2006; Wang et al. 2014). Fang and Liu (2002) and Jiang et al. (2013) suggested acidic pH as a predominant condition for VFA optimization. Nevertheless, some studies (Cai et al. 2009; Jie et al. 2014; Garcia-Aguirre et al. 2017) have demonstrated that alkaline pH could promote higher VFA production than acidic pH. Moreover, depending on the downstream application, proper tuning on the working pH of acidogenic fermentation process should be considered in order to promote VFA generation and to adjust the desired individual VFA percentage in the fermentation broth. If the fermentation effluent is sent to produce PHA, acetate and butyrate tend to form hydroxybutyrate (HB) monomers, whereas the presence of propionate and valeric lead to the formation of hydroxyvalerate monomers (Bengtsson et al. 2008; Jankowska et al. 2015). Poly-3-hydroxybutyrate (PHB) and poly-3-hydroxyvalerate (PHV) biopolymers have very distinct characteristics in terms of heat resistance, elasticity, durability, and transparency, among others (Chanprateep et al. 2010; Chee et al. 2010; Bugnicourt et al. 2014).

The objective of this study was to evaluate VFA production in acidogenic fermentation using (i) source-sorted OFMSW from a full-scale mechanical-biological treatment (MBT) plant in the metropolitan area of Barcelona, and (ii) FW from a university canteen. By considering the adjustment of pH value, a comparison between individual VFA compositions (acetic, propionic, butyric, and valeric acids) was assessed to observe the effect of changes when mixed microbial cultures were used.

## Materials and methods

### Substrate and inoculum

The source-sorted OFMSW used in this research was collected from a MBT plant in the metropolitan area of Barcelona. In this plant, the source-sorted OFMSW was pre-treated to remove certain undesired materials and to perform basic operations, such as particle size reduction, adjustment of the water content of the feedstock to the wet AD process, and removal of precipitable inerts (e.g. sand, clay, and glass) and floating materials (e.g., plastics) as described in Astals et al. (2013). It is important to highlight that in this MBT plant, the total solids (TS) adjustment was performed by using the liquid fraction of anaerobically digested OFMSW of the same plant, which provides extra alkalinity to this substrate (Gottardo et al. 2017), but also a high NH_4_^+^–N content. Once it was collected, the pre-treated OFMSW was kept in the refrigerator of laboratory at 4 °C until its use. Table 1 shows the main characteristics of the collected OFMSW in three collection periods (A1, A2, and A3).

**Table 1:** Characteristics of the OFMSW used in this study in every collection period

Parameter	Units	Period A1	Period A2	Period A3
Total solids (TS)	% *w*/*w*	6.60 ± 1.46	5.63 ± 0.76	6.41 ± 0.59
Volatile solids (VS)	% *w*/*w*	5.15 ± 1.25	4.18 ± 0.63	4.87 ± 0.53
Soluble COD (sCOD)	g L^−1^	72.39 ± 11.15	72.75 ± 18.47	57.50
Volatile fatty acids (VFAs)	g L^−1^	10.12 ± 0.90	8.61 ± 0.78	9.62 ± 1.57
Alkalinity	g CaCO_3_ L^−1^	4.99 ± 0.51	–	4.81 ± 0.18
TAN	g NH_4_^+^–N L^−1^	–	2.84 ± 0.85	2.31 ± 0.54
pH	–	6.06 ± 0.11	6.75 ± 0.11	5.85 ± 0.04

The other substrate used in this research was FW collected from a university canteen. In order to minimize the great variability that could be found in FW, the collection of this organic waste was performed approximately every 14 days at the end of the week and immediately blended with deionized water and shredded (Bosch, MMB66G5M) in the laboratory. Minimum amount of water was added in this step in order to obtain a concentrated feedstock. The shredded FW was stored in refrigerator at 4 °C. When it was needed for feeding, proper quantity of deionized water was added to dilute it and to control the total solids contents. The main characteristics of FW are summarized in Table 2 according to its collection periods (B1 to B8).

**Table 2:** Characteristics of the FW used in this study in every collection period

Parameter	Units	Period B1	Period B2	Period B3	Period B4	Period B5	Period B6	Period B7	Period B8
Total solids	% *w*/*w*	4.3 ± 0.7	5.9	4.3 ± 0.6	5.0*	5.6 ± 1.7*	7.3 ± 1.1*	7.3 ± 0.3*	6.6 ± 0.5*
Volatile solids	% *w*/*w*	4.1 ± 0.7	5.6	4.1 ± 0.6	4.1*	5.2 ± 1.7*	6.1 ± 0.8*	5.7 ± 0.2*	5.5 ± 0.4*
Soluble COD	g L^−1^	15.8 ± 12.7	32.4	36.7 ± 13.3	23.6 ± 6.7	40.1 ± 6.8	37.0 ± 2.9	38.7 ± 4.3	32.3 ± 14.7
Volatile fatty acids	g L^−1^	1.0 ± 0.4	1.9 ± 0.4	1.4 ± 0.3	0.7 ± 0.1	1.1 ± 0.2	1.0 ± 0.0	1.2 ± 0.1	1.5 ± 0.2
TAN	mg NH_4_^+^–N L^−1^	–	153.0	81.0 ± 24.6	48.7	15.5 ± 4.5	34.1 ± 4.8	50.4 ± 1.3	26.7 ± 6.2
pH	–	4.8 ± 0.1	4.4 ± 0.4	4.2 ± 0.1	4.2 ± 0.1	5.7 ± 1.3**	5.8 ± 0.3**	6.6 ± 0.8**	6.5 ± 0.8**

*TS and VS analyzed after the addition of external alkalinity (NaHCO_3_)

**pH measurement in periods B5 to B8 was performed after the addition of NaHCO_3_

In the start-up of semi-continuous acidogenic fermenters treating OFMSW, the inoculum used was obtained from a lab-scale acidogenic fermenter treating residual organic matter (ROM) (Dosta et al. 2018). The effluent of the continuous stirred-tank reactor treating OFMSW at pH 6 was used as inoculum to start up the lab-scale fermenters treating FW. The effluent of the fermenter treating FW at pH 6 was used to inoculate batch tests of FW.

### Semi-continuous acidogenic fermenters

Two jacketed lab-scale reactors with an effective working volume of 4.5 L and mechanically stirred (using IKA-Werker, RW 16 basic functioning at approximately 170 rpm) were used as fermentation reactors at mesophilic conditions working with source-sorted OFMSW and FW. These fermenters were initially operated to test the influence of pH in the acidogenic fermentation process of OFMSW at an HRT of 3.5 days (Dosta et al. 2018) working at (i) acidic pH near 6 (namely, 5.63–6.34) and (ii) alkaline pH near 10 (namely, 9.82–10.01). In fermenter working with OFMSW under acidic conditions, no external chemical addition was needed for pH control due to the buffer capacity of the collected OFMSW. When treating the same substrate at pH 10, concentrated sodium hydroxide (NaOH) solution (10 M) was dosed manually once per day right after the feeding was performed. To start up the fermenters, the inoculum was kept stirring for 24 h under mesophilic conditions (35 °C) to acclimate again the inoculum (which was previously stored at 4 °C for about 4 weeks). The equivalent quantity of substrate (OFMSW) was fed manually to fermenters once per day (fed-batch culture). The effluent of fermenters were characterized every day except weekend. To avoid a pressure drop inside the fermenters and the entrance of air during the draw-off operation, nitrogen gas was flushed during effluent extraction and substrate feeding operations.

During the operation of the fermenters treating OFMSW, another lab-scale fermenter (4.5 L) was set up to investigate the possible inhibition due to organic loading rate (OLR) and VFA. This fermenter was fed with OFMSW and worked at pH near 6 without external chemical addition. When the production of VFA was stable, diluted OFMSW with deionized water at 50% in volume was fed to check if the specific production of VFA per volatile solids (VS) fed was maintained. Finally, undiluted OFMSW was used again as feeding.

After these experiments with OFMSW, purged biomass from the fermenter working at pH 6 was used to inoculate two new fermenters fed with FW. The acidogenic fermenters of FW were operated under the same operational conditions as for OFMSW: HRT of 3.5 days, once per day feeding, mesophilic temperature (35 °C), and at acidic and alkaline pH (near 6 and 10, respectively). Alkalinity is a key control parameter to operate digesters in the optimum pH conditions for VFA production (Ratanatamskul and Saleart 2016), but the poor alkalinity of the FW was unable to avoid a high pH descent during fermentation. To overcome this problem, NaHCO_3_ was added since it increases buffer capacity and it is partially basic when dissolved in water. However, if NaHCO_3_ was only used for pH adjustment, especially in fermenter working at pH 10, high quantities would be needed. For this reason, strong alkaline (NaOH) addition was combined with NaHCO_3_ dosage. In this study, depending on the difference between the pH of reactor and its pre-set value, different doses of NaHCO_3_ (5, 10, or 15 g L^−1^) was added to the FW: 5 g L^−1^ when pH_set point_ − pH_effluent_ was ≤ 0.1; 10 g L^−1^ when pH_set point_ − pH_effluent_ was ≤ 0.2; and 15 g L^−1^ when pH_set point_ − pH_effluent_ was ≤ 0.5.

### Batch fermentation tests

Batch tests were carried out to assess the influence of pH on the production and distribution of VFA at short-term conditions. Identical serum bottles with a working volume of 200 mL were filled with inoculum and substrate according to their volatile solids contents to obtain a ratio of 1:1 by weight. This value was similar to the one used by Ji et al. (2010), but instead of using a ratio based on volatile suspended solids (VSS) content, in this study, VS basis was applied. Each condition evaluated in the batch tests was performed by duplicate and the duration was set at 10 days based on Garcia-Aguirre et al. (2017) results and considering that the HRT of the semi-continuous fermenters (3.5 days) was almost triplicated. These bottles were placed in an incubator (Memmert, Pass-through ovens UF750) running at 35 °C and VFA analysis was performed every day from day 0 to 7 and on day 10 to check the progress of VFA production. According to the pH conditions (pH 4, 6, 7.5, 9, 10, 11, and uncontrolled), concentrated solutions (10 M) of hydrochloric acid (HCl) and NaOH were used to adjust pH to their pre-set value. The measurement and adjustment of pH were performed on the days when VFA analysis was carried out. After that, each bottle was flushed with nitrogen gas for 1 min to avoid the entrance of air in the headspace and was closed with PTFE-Butyl septum.

### Analytical methods

TS, VS, and soluble chemical oxygen demand (sCOD) were analyzed in accordance with the standard methods 2540B, 2540E, and 5220D, respectively (APHA 2012). Alkalinity was determined through titration method (0.2 mL per 10 s) until pH 4.3 by using pH-Burette 24 (Crison) with 0.1 M HCl. The pH of reactor was measured with pressurized gel-electrolyte electrodes (Mettler Toledo, HA405-DPA-SC-S8/225). For total ammonium nitrogen (TAN) concentration determination, sample was centrifuged at 4,000 rpm for 15 min, the supernatant was filtered through 0.45-μm pore size regenerated cellulose syringe filter. Proper dilution factor was applied to have it in between 1 and 100 ppm which was determined using high-performance ammonium ion selective electrode (Thermo Scientific, Orion 9512HPBNWP). Free ammonia nitrogen (FAN) concentration was calculated considering a dissociation constant for the ammonium ion (pK_a_) of 8.95 at 35 °C (Yun et al. 2016). For VFA analysis, the filtered sample was acidified with 85% phosphoric acid and diluted 10-fold. VFA was measured using gas chromatograph (Shimadzu, GC-2010 plus) equipped with capillary column (Nukol™, 15 m × 0.53 mm × 0.5 μm) and flame ionization detector (FID). Initially, the temperature of capillary column was 80 °C, it was heated by 10 °C per minute to 110 °C followed by 15 °C per minute to 145 °C and then by 20 °C per minute to 190 °C. The temperatures of injector and detector were 280 °C and 300 °C, respectively. Helium was carrier gas, hydrogen was fuel gas, and synthetic air was oxidizing gas. Acetic, propionic, isobutyric, butyric, isovaleric, valeric, isocaproic, caproic, and heptanoic acids were those VFA which could be detected by the programmed method for this gas chromatograph.

## Results and discussion

### Effect of pH in the acidogenic fermentation of OFMSW

Table 3 shows the main characteristics of the obtained fermentation effluent in both digesters in different collection periods of the OFMSW. As observed in Table 3, the acidogenic fermentation of this substrate increased its VFA concentration but not in a large extent: an increase of 13.9–16.9% and 9.8–15.3% was recorded at acidic and alkaline conditions respectively. This was related to the fact that the source-sorted OFMSW used in this study came from a MBT plant of the Barcelona Metropolitan Area and it had a high concentration of VFA, in the range of 8.61–10.12 g L^−1^ (see Table 1).

**Table 3:** Characteristics of the obtained effluent for the OFMSW acidogenic fermentation process under mesophilic conditions (35 °C) at pH 6 and 10

Parameters	Units	Mesophilic fermenter at acidic pH (6)			Mesophilic fermenter at alkaline pH (10)		
		Period A1	Period A2	Period A3	Period A1	Period A2	Period A3
Alkalinity	g CaCO_3_ L^−1^	5.28 ± 0.14	–	5.44 ± 0.15	9.83 ± 0.65	9.00	10.60 ± 0.87
TAN	g NH_4_^+^–N L^−1^	–	3.07 ± 0.10	3.21 ± 0.07	–	2.32 ± 0.63	1.61 ± 0.40
Free ammonia	mg NH_3_–N L^−1^	–	7.52 ± 0.31	1.54 ± 0.04	–	2134.13 ± 745.10	1418.63 ± 453.16
pH	–	5.98 ± 0.26	6.34 ± 0.21	5.63 ± 0.05	9.90 ± 0.16	10.01 ± 0.15	9.82 ± 0.09
sCOD	g L^−1^	78.37 ± 10.94	67.73 ± 9.48	–	92.72 ± 28.89	83.48 ± 18.94	–
COD_VFA_/sCOD	%	23.00 ± 4.19	23.20 ± 3.64	–	16.51 ± 2.81	19.78 ± 4.05	–
VFA	g L^−1^	11.53 ± 0.53	9.75 ± 0.94	11.25 ± 0.62	11.11 ± 0.52	9.71 ± 1.03	11.09 ± 1.68
(C_2_ + C_4_)/(C_3_ + C_5_)	–	1.67 ± 0.22	1.89 ± 0.08	2.00 ± 0.13	1.91 ± 0.07	1.90 ± 0.10	2.21 ± 0.15
Acetic acid	%	35.27 ± 4.68	40.73 ± 1.68	38.23 ± 3.32	38.68 ± 1.13	41.17 ± 1.25	41.11 ± 2.41
Propionic acid	%	22.05 ± 2.34	20.75 ± 1.12	18.23 ± 0.36	20.06 ± 0.41	20.88 ± 1.00	17.98 ± 0.89
Butyric acid	%	19.79 ± 1.42	16.60 ± 1.32	20.48 ± 2.18	16.36 ± 0.31	13.04 ± 1.98	18.63 ± 2.65
Valeric acid	%	11.60 ± 1.14	9.64 ± 0.84	11.90 ± 1.38	8.25 ± 0.20	7.19 ± 0.63	8.76 ± 1.54
Other acids	%	11.29 ± 2.40	12.28 ± 1.24	11.16 ± 1.81	16.65 ± 0.51	17.72 ± 1.22	13.52 ± 1.87

**Fig. 1:**
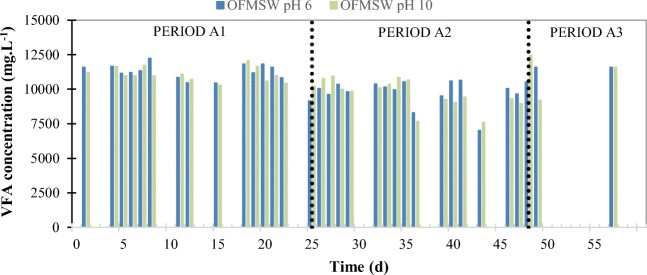
VFA production in mesophilic acidogenic fermenters treating OFMSW at acidic and alkaline pH (6 and 10, respectively)

Figure 1 shows the evolution of the total VFA concentration in the effluents of fermenters working at pH 6 and 10. Due to VFA production, a drop of pH until values of 5.63–6.34 was obtained in the fermenter working under acidic conditions without external control. Although some studies indicate that higher VFA production could be obtained under alkaline conditions than under acidic conditions (Lee et al. 2014; Garcia-Aguirre et al. 2017), in this study, the results showed that the VFA production under pH 10 was not as high as what could be expected, reaching a similar VFA production and distribution of individual VFAs to the obtained at pH 6.0 (see Table 3). As stated in Table 3, the sCOD was higher in the fermenter working at pH 10 than in the fermenter working at lower pH, because hydrolysis was favored at the alkaline pH (Li et al. 2018; Liu et al. 2018; Zou et al. 2018). In alkaline pH, a strong repulsion exists between extracellular polymeric substances (EPS) followed by release of carbohydrate and protein from internal cell to the environment (Yu et al. 2008; Feng et al. 2014; Yuan et al. 2015). The increase of sCOD at pH 10 was in the range of 14.6–28.0 which is in agreement with results obtained by other authors despite the type of substrate (Yu et al. 2008; Marin et al. 2010; Yuan et al. 2015), varying between 20% and 49-fold. Although an enhanced solubilization of organic matter was reported with respect to the fermenter working at pH near 6 (18.2–23.2% higher), under alkaline conditions, it was not observed an increase of the ratio COD_VFA_/sCOD with respect to the reactor working under acidic conditions, since the solubilization of COD did not significantly impact in a higher production of VFA. Besides, the average NH_4_^+^–N concentration of the reactor working at pH 6 (3.07–3.21 g NH_4_^+^–N L^−1^) was similar to that obtained in the OFMSW fed, but it was lower in the reactor working at pH 10 (between 2.32 and 1.61 g NH_4_^+^–N L^−1^) due to ammonia stripping. Furthermore, the much higher free ammonia concentration inside the reactor working at pH 10 may lead to a strong inhibition of acidogenic bacteria (Bai et al. 2017). In fact, ammonium concentration over 5000 mg L^−1^ has been reported as toxic to anaerobic bacteria, including acidogens (Yu and Fang 2001; Lee et al. 2014), although nitrogen is essential for biomass growth.

**Fig. 2:**
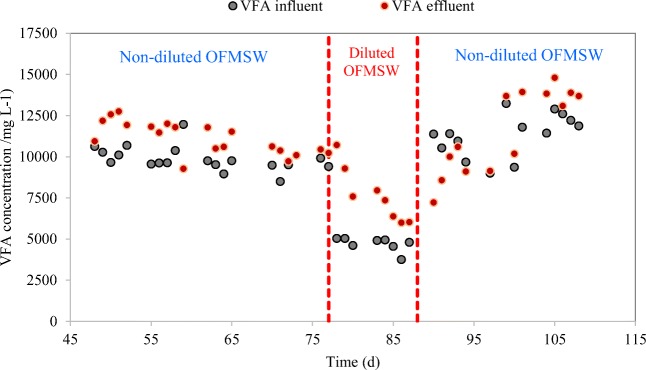
Inhibition assay of VFA production using diluted and non-diluted OFMSW (collected in period A3)

Jiang et al. (2013) studied the OLR effect on VFA production and showed that the reactor’s operation when using FW at a high OLR of 15.5 g VS (L day)^−1^ was unstable because the fermentation broth was very viscous. This led to some undesirable fluctuations during the experiment and caused difficulties on the way to find out the optimal process conditions, especially in the batch tests. In the present study, the experiment was carried out at roughly 17.5 g VS (L day)^−1^ which was higher than the value reported by Jiang et al. (2013). To clarify if the low increase of VFA was due to high OLR or VFA inhibition, an additional experiment was performed in a separate semi-continuous fermenter working at pH near 6 without external chemical control. In this reactor, undiluted OFMSW (from period A3) was fed for 30 days until reaching a constant operation (see Fig. 2), and afterwards diluted OFMSW with deionized water at 50% *v*/*v* was used as substrate, observing a consequent decrease of the obtained VFA concentration and a tendency to reach the same ratio of total VFA_effluent_/VS_influent_. After a 10-day feeding with diluted OFMSW, the digester was fed again with undiluted OFMSW reaching VFA concentrations in the range of the stage before the dilution. Hence, this assay proved that there was not inhibition due to OLR or VFA since the VFA production during the dilution period decreased with time and became approximately half of that without dilution.

**Fig. 3:**
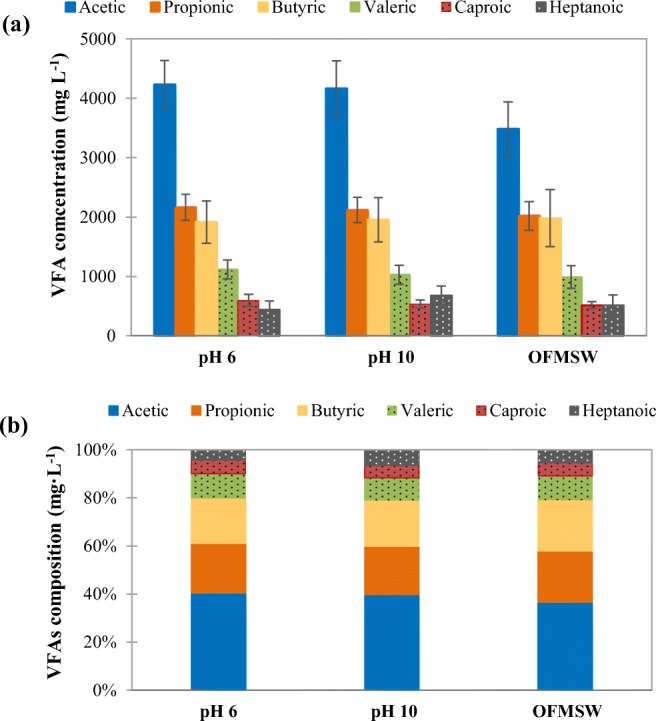
Average concentration of individual VFA (**a**) and their composition (**b**) in the influent and effluents of acidogenic fermenters of OFMSW at pH 6 and pH 10 (considering all the experimental period)

Figure 3 shows the average concentrations of individual VFA of the influent (OFMSW) and in the effluents of both fermenters (pH 6 and pH 10). Unexpectedly, the average concentration of each particular VFA was very similar between feeding and effluent. By comparing their composition in percentage, the differences were only in small extent. Due to the high concentration of VFA in the feeding, after acidogenic fermentation, only a small increment of VFA concentration was detected in the effluent. Regarding these results, the OFMSW collected from MBT plant could be pre-fermented if its processing might fulfill the required conditions for hydrolytic and acidogenic activity. Prefermentation of OFMSW could be performed not only by the inherent microorganisms present in this biowaste, but also by the hydrolytic and acidogenic microorganisms present in the recirculated AD supernatant (mesophilic conditions) during the pre-treatment process (with duration of several hours). However, to better understand this prefermentation process in the MBT plant, a detailed study should be carried out with the aim to investigate if there is any special pre-treatment unit after the arrival of fresh OFMSW and before its feeding to anaerobic digestion where acidogenic fermentation takes place.

To sum up, the overall increase of VFA concentration was lower than 20% based on the VFA concentration of substrate. As a result, the ratio (C_2_ + C_4_)/(C_3_ + C_5_) was in the range of 1.7–2.2 and did not give much change based on that ratio of the influent. The effluent had considerable amount of total VFA concentration (9.8–11.5 g L^−1^) with a proper distribution among acetic, propionic, butyric, and valeric acids (namely, C_2_ 36–41%, C_3_ 18–22%, C_4_ 13–21%, and C_5_ 7–12%), to be used as feedstock of PHA production. Table SI (in the Supplementary Material) summarizes a complete individual VFA composition on COD basis. Finally, the ratio of VFA with respect to the soluble COD (COD_VFA_/sCOD) in the fermentation effluent was lower under alkaline conditions at pH 10 (16.5–19.8) than under acidic conditions at pH 6 (23.0–23.2) due to a higher hydrolysis of organic matter under alkaline conditions (Yuan et al. 2015).

### Effect of pH in the acidogenic fermentation of FW

#### Batch fermentation tests using FW

**Fig. 4:**
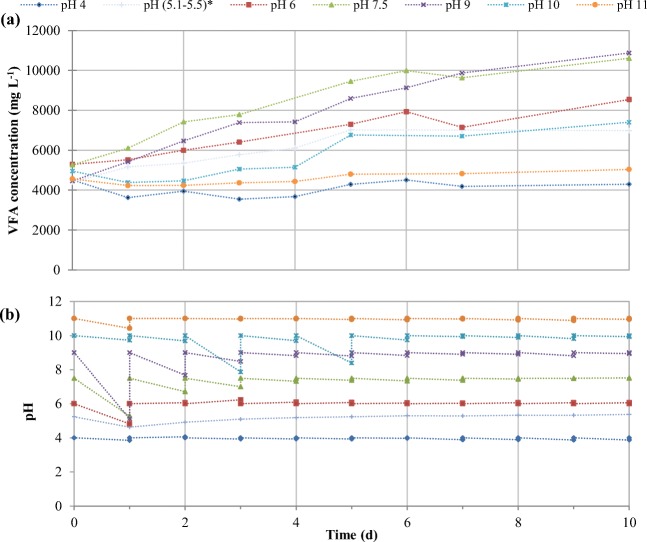
Average VFA concentration (a) and pH control evolution (b) in the fermentation batch tests of FW at different pH conditions

Considering the prefermentation observed in the OFMSW collected in a MBT plant and the possible free ammonia inhibition of acidogenic fermentation working under alkaline conditions, the feedstock of the acidogenic fermenters was substituted for FW which is considered one of the major components of OFMSW and is usually characterized by a low nitrogen-to-carbon ratio (Mu et al. 2017). To study the effect of pH on the acidogenic fermentation of FW at short-term conditions, several batch tests under pH conditions between 4 and 11 were carried out as stated in the “Batch fermentation tests” section (using FW and inoculum of the lab-scale fermenters during period B6). Figure 4 shows the profiles of VFA production and pH of these batch tests, which is in accordance to that obtained by Zheng et al. (2018), who reported an optimum pH of 8.0 when treating kitchen wastewater at 21 ± 1 °C. As observed in this figure, at pH 9 and 7.5, the increment of VFA concentration was approximately doubled to 10.9 g L^−1^ and 10.6 g L^−1^, respectively, on day 10 with respect to their initial values. Zhang et al. (2005) also reported a higher rate of hydrolysis and acidogenesis of kitchen waste at neutral pH (7) with respect to lower (5) or higher (10, 11) pH values. VFA production in 10 days was also high at pH 6 (8.5 g L^−1^), pH 10 (7.4 g L^−1^), and pH between 5.1 and 5.5 without external chemical addition (7.0 g L^−1^). However, at pH 4 and 11, VFA productions were very limited and became the lowest among all, demonstrating that under these extreme values of pH, the production of VFA was not favored since acidogenic bacteria cannot survive under extremely acid (pH 3) or alkaline (pH 12) conditions (Strazzera et al. 2018).

Regarding pH profiles, on the first 3 days, the assays with higher VFA production, namely at pH 6, 7.5, and 9, experimented some pH fluctuations and pH decreased drastically from 6.0 to 4.8, from 7.5 to 5.3, and from 9.0 to 5.1, although every day the pH was adjusted to the set point. These sudden pH drops could affect the production of VFA and even the composition of VFA generated in the following days. In fact, at the beginning of the batch test, acidogenic bacteria were undergoing acclimation with new environment (different pH medium) and in this adaption period, there are normally some fluctuations and instability of fermentation broth (Garcia-Aguirre et al. 2017). However, after the third day, pH variations in the batch became small, within the range of ± 0.3 from their pre-set values. Nonetheless, continuous generation of VFA and existence of high concentration of soluble organics (Lissens et al. 2004; Zhang et al. 2005) were pulling the equilibrium to a partially acidic pH. This was proven when the conditions of pH 6 and uncontrolled pH only dropped during the first day of this experiment and then they increased very slowly day to day afterwards as compared to pH 7.5 and pH 9 which reduced with a slightly faster rate.

**Fig. 5:**
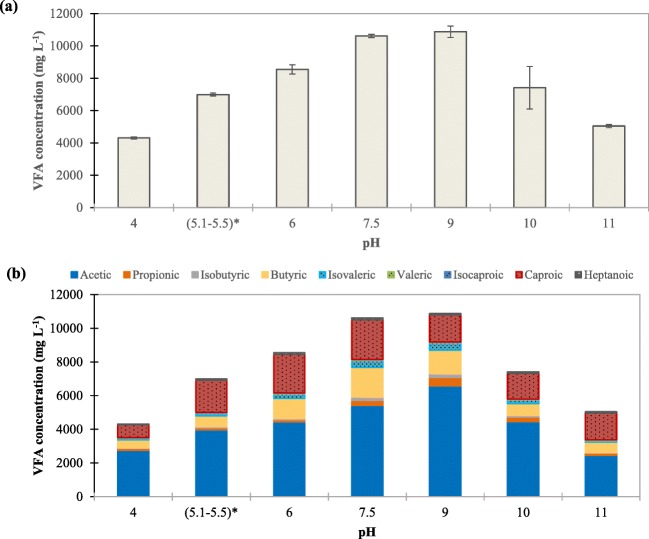
Total VFA concentration (**a**) and average individual VFA concentration (**b**) at 10 days of the fermentation batch tests of FW at different pH conditions

pH is a well-known operational parameter which can affect the production of VFA and its composition since it has great influence on hydrolysis and acidogenic fermentation (Atasoy et al. 2018; Begum et al. 2018; Zhao et al. 2018). At the same time, it can also lead to various metabolism pathways to produce different concentrations of individual VFA (Zhou et al. 2018). Figure 5 shows the composition of each individual acid on day 10. In the tests carried out at highly acidic (pH 4) or alkaline (pH 11) conditions, the final concentration and distribution of VFA were very affected by the initial conditions of the experiment. Among the rest of the tests, the highest percentage of acetic acid was recorded at pH 9 and 10, with 60.5% and 60.2% of acetic acid, respectively. Lower pH values yielded lower proportions of acetic acid in the individual VFA distribution: 56.7% (pH 5.1–5.4), 52.0% (pH 6), and 51.1% (pH 7.5). This is consistent with the results of Zhang et al. (2005), who also observed that pH 9 and 11 intensely favored acetic acid production over other VFAs in comparison to the fermentation broth obtained at pH 5 and 7 when treating kitchen waste at 35 °C.

Caproic and butyric acids were also produced considerably in the acidogenic fermentation tests and changed when the operating pH increased. Caproic acid production in the biorefinery context still has space for development since there are only few studies published related to this short-chain fatty acids. At the two pHs close to neutrality (pH 6 and pH 7.5), approximately 15% of butyric and 20% of caproic acids were produced in 10 days. For butyric acid production, it went downwards when the pH decreased to 5.1–5.5 (9.3%) as well as when the pH increased to 10 (9.5%). These results were in accordance with those from Jiang et al. (2013). For caproic acid production, the concentration changed without showing a clear and predictable shift. The highest concentration of VFA was produced at pH 9, but most of them was acetic acid with 60.5%, being butyric and caproic acids at 12.8% and 14.8% respectively. Minorities like propionic (4.6%), isovaleric (4.0%), and isobutyric (2.0%) were also detected. Increasing from pH 9 to 10, not much difference in acetic (60.2%) was observed, butyric (9.5%) was roughly 3% lesser, and more proportion of caproic acid (20.8%) was produced.

To sum up, more butyric acid was generated at pH 6 and 7.5 and it decreased when the pH was raised. More than 48% of VFA produced was acetic acid, and its highest percentage was found at pH 9 and pH 10. As a result, it can be concluded that the pH condition affected not only the VFA production, but also its composition. However, it should be noted that these experiments were performed at short-term conditions using an inoculum used to work under pH 6.0 (which already had an initial VFA content), so a general tendency could be observed but should be confirmed under long-term operation.

#### Semi-continuous acidogenic fermenters using FW

The characterization of FW in the periods analyzed in this study (based on its collection from periods B1 to B8) is shown in Table 2. TS concentration was approximately 4.3% *w*/*w* in the first period (B1) with more than 95% being volatile solids (VS). In the third working period (B3), similar solid contents were registered, but in periods B4 to B8, slightly higher TS (ranging from 5.0 to 7.3%) and VS (ranging from 4.1 to 6.2%) were recorded (in these periods, the TS content was calculated after external addition of NaHCO_3_, and consequently approximately 80% of them were VS). As mentioned in the “Substrate and inoculum” section, VS was adjusted in the feeding by adding deionized water, and its value was maintained between 4.1 and 6.1%. The initial VFA concentration of FW was low, ranging from 0.7 to 1.9 g L^−1^; TAN concentration was lower than 160 mg NH_4_^+^–N L^−1^; and its pH was in the range of 4.2–5.8.

To start-up the acidogenic fermenters, the fermentation-mixed liquor, which was previously treating OFMSW at pH 6, was used to inoculate two new semi-continuous reactors for the fermentation of FW. Due to the characteristics of FW, pH was adjusted both at pH 6 and 10 using NaHCO_3_ and NaOH (as stated in the “Materials and methods” Section), although co-fermentation of the collected OFMSW of this study and FW could also be considered to balance the unfavorable TAN concentration of OFMSW while reducing or even avoid the external alkalinity needs for FW. In fact, some studies have reported successful co-fermentation of FW and OFMSW at pilot scale under acidic conditions compensating the deficit of alkalinity of these substrates with other feedstock such as waste activated sludge (Garcia-Aguirre et al. 2019; Valentino et al. 2019).

Table 4 and Table 5 show the composition of effluents of semi-continuous fermenters working at pH 6 and 10, respectively. Table SII and SIII (in the Supplementary Material) show the complete VFA distribution analysis in COD basis for both fermenters. At the beginning of this experiment, the TS and VS decreased gradually in both fermenters since FW in period B1 had slightly lower TS and VS percentages than OFMSW. Most fluctuations were observed in period B1 when there was changing of substrates from OFMSW to FW. During this start-up stage, there was still remaining unfermented or insoluble organic matter from OFMSW. Adding FW into this liquid mixture might prompt unstable broth with high remaining organic matter which may provoke negative impacts to the system (Mata-Alvarez et al. 2014). FW is usually characterized by a low nitrogen-to-carbon ratio and, consequently, TAN in the effluent during period B1 and from B3 to B8 was below 700 mg NH_4_^+^–N L^−1^ at both pH 6 and pH 10. However, the collected FW in period B2 contained a significant quantity of meat residues which represented an important source of proteins to the acidogenic fermentation process, leading to an unusual peak of TAN in the effluent.

**Table 4:** Characteristics of the FW semi-continuous fermenter effluent at acidic pH (6) under mesophilic conditions

Parameters	Units	Effluent at pH **6**							
		Period B1	Period B2	Period B3	Period B4	Period B5	Period B6	Period B7	Period B8
Alkalinity	g CaCO_3_ L^−1^	–	–	–	0.78 ± 0.78	1.97 ± 0.30	3.22 ± 0.67	–	–
TAN	mg NH_4_^+^–N L^−1^	473	1075	652 ± 288	440	618 ± 272	517 ± 42	670 ± 40	390 ± 210
Free ammonia	mg NH_3_–N L^−1^	0.05	1.29	0.51 ± 0.29	0.38	0.36 ± 0.20	0.39 ± 0.04	0.61 ± 51.43	0.39 ± 0.27
pH	–	4.95 ± 0.28	6.03 ± 0.56	5.84 ± 0.53	5.89 ± 0.60	5.71 ± 0.33	5.83 ± 0.27	5.91 ± 0.16	5.95 ± 0.20
sCOD	g L^−1^	56.42 ± 35.39	38.08	43.58 ± 11.51	36.17 ± 0.17	44.74 ± 6.87	48.09 ± 3.68	49.92 ± 2.51	48.53 ± 1.62
COD_VFA_/sCOD	%	12.98 ± 9.70	59.12	23.55 ± 5.24	14.60 ± 1.94	20.82 ± 4.10	22.28 ± 3.08	26.83 ± 2.40	29.50 ± 1.81
VFA	g L^−1^	4.66 ± 1.13	11.73 ± 2.37	4.74 ± 2.31	3.65 ± 0.67	5.46 ± 0.46	7.34 ± 0.85	9.60 ± 0.57	9.42 ± 1.26
(C_2_ + C_4_)/(C_3_ + C_5_)	–	3.68 ± 5.22	5.82 ± 5.65	9.74 ± 5.62	21.61 ± 10.15	15.49 ± 2.46	18.77 ± 2.25	20.32 ± 2.80	21.91 ± 7.00
Acetic acid	%	63.61 ± 17.58	32.30 ± 2.86	49.63 ± 8.70	56.72 ± 2.99	53.80 ± 12.18	56.10 ± 9.24	60.34 ± 3.73	59.00 ± 2.81
Propionic acid	%	10.11 ± 6.25	8.50 ± 2.21	3.33 ± 1.77	1.17 ± 1.54	1.81 ± 0.19	1.30 ± 0.10	1.22 ± 0.11	0.96 ± 0.57
Butyric acid	%	11.46 ± 4.40	32.42 ± 1.17	11.51 ± 1.60	11.87 ± 1.10	12.63 ± 3.35	11.72 ± 1.87	12.92 ± 1.90	11.40 ± 1.41
Valeric acid	%	7.50 ± 2.44	9.98 ± 0.90	4.60 ± 1.33	2.46 ± 0.37	2.66 ± 0.59	2.33 ± 0.43	2.42 ± 0.31	2.49 ± 0.32
Other acids	%	7.32 ± 2.93	16.80 ± 1.79	30.93 ± 3.35	27.78 ± 1.50	29.1 ± 4.08	28.55 ± 2.91	23.1 ± 1.51	26.15 ± 1.28

**Table 5:** Characteristics of the FW semi-continuous fermenter effluent at alkaline pH under mesophilic conditions

Parameters	Units	Effluent at alkaline pH (with control to pH 10)			
		Period B1	Period B2	Period B3	Period B4
Alkalinity	g CaCO_3_ L^−1^	–	–	–	5.81 ± 0.26
TAN	mg NH_4_^+^–N L^−1^	–	875.0	445.3 ± 83.8	394.0
Free ammonia	mg NH_3_–N L^−1^	–	67.2	354.0 ± 85.7	256.3
pH	–	8.64 ± 0.97	7.87 ± 0.75	9.54 ± 0.46	9.22 ± 0.32
sCOD	g L^−1^	61.42 ± 42.47	44.87	49.51 ± 2.52	41.97 ± 4.95
COD_VFA_/sCOD	%	14.40 ± 11.32	31.42	20.50 ± 4.84	19.26 ± 1.98
VFA	g L^−1^	5.24 ± 1.50	9.33 ± 1.75	7.92 ± 4.93	6.19 ± 1.38
(C_2_ + C_4_)/(C_3_ + C_5_)	–	3.85 ± 1.83	13.01 ± 2.39	14.49 ± 6.48	20.14 ± 2.53
Acetic acid	%	56.39 ± 12.81	81.55 ± 1.85	83.75 ± 7.64	90.63 ± 0.82
Propionic acid	%	12.24 ± 4.48	2.77 ± 0.64	2.26 ± 0.73	1.96 ± 0.25
Butyric acid	%	16.14 ± 4.08	8.21 ± 1.56	6.90 ± 2.81	3.84 ± 0.25
Valeric acid	%	7.61 ± 2.57	3.68 ± 0.44	4.18 ± 0.97	2.74 ± 0.28
Other acids	%	7.62 ± 2.25	3.79 ± 0.53	2.91 ± 0.77	0.83 ± 0.06

**Fig. 6:**
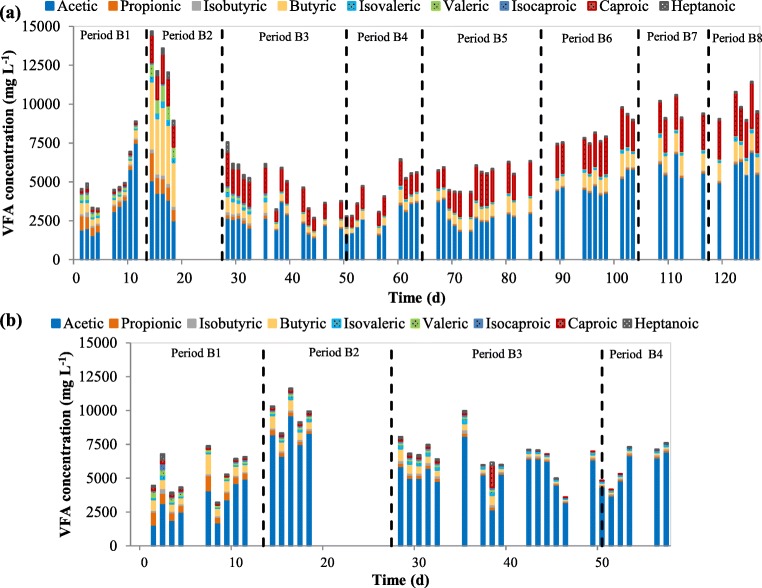
Accumulative individual VFA of acidogenic fermentation at mesophilic conditions using FW for acidic pH near 6.0 (**a**) and alkaline pH near 9.5–10 (**b**)

Figure 6 panels a and b show the production of VFA of FW at pH 6 and pH 10. The fermenter working at pH 6 had an operating period of 126 days, while for pH 10, it was 57 days. As observed in this figure, the VFA production in the acidogenic fermentation of FW was distinct from one period to another. In this regard, it should be taken into account that this process was started up from the reactor treating OFMSW, so the microbial cultures of this reactor were adapted to the new substrate. Therefore, period B1 of anaerobic fermentation of FW can be considered a start-up period for semi-continuous acidogenic fermentation of FW. Due to the heterogeneity of FW, when the substrate collected changed from one period to another, the shift in acid distribution was noticed.

In the second period (period B2), the highest production was observed, reaching an average VFA concentration of 11.73 ± 2.37 g VFA L^−1^ at pH 6 and 9.33 ± 1.75 g VFA L^−1^ at alkaline pH, which were almost the double of that obtained in period B1. It is important to highlight that during period B2, the effluent pH in alkaline conditions was 7.9 ± 0.8; therefore, a lower production of VFA could be related to the difficulties to remain pH value near 10. When using FW from the second collection (period B2), butyric acid (32.7%) became competitive with acetic acid (31.9%) and this could not be seen in the any other period. Considerable amount of protein-rich organic waste in FW collected during period B2 could be related to a higher production of both acetic and butyric acids (Feng et al. 2014). Percentages of propionic acid (8.1%) and valeric acid (10.3%) progressively decreased, which affected the ratio between odd and even carbons in the VFAs, a parameter that should be taken into account if the VFAs are to be used in PHA production.

**Fig. 7:**
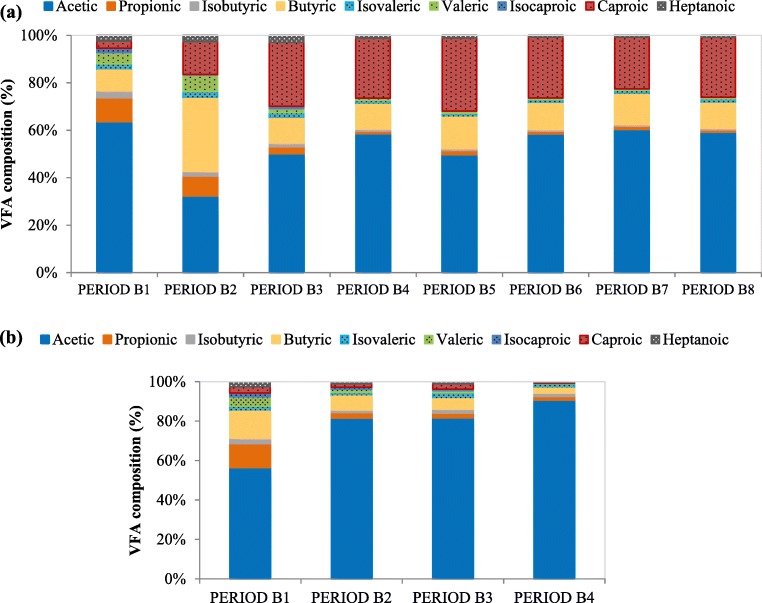
Average VFA distribution of the liquid effluent of FW fermentation at different periods for acidic pH near 6.0 (**a**) and alkaline pH near 9.5–10 (**b**)

During the third period in which the FW had a lower VS content than in the previous period, VFA production at alkaline pH between 9.5 and 10 (7.92 ± 4.93 g VFA L^−1^) was always higher than that at pH 6 (4.74 ± 2.31 g VFA L^−1^). This tendency remained the same during period B4 when pH was between 9.2 and 10, which is consistent with the short-term pH effect observation during fermentation batch tests and the findings of Park et al. (2014) who reported that alkaline pH can improve hydrolysis of organic matter and provide readily fermentation substrate for acidogenic bacteria for VFA production. In fact, Zheng et al. (2013) reported an increased functional bacteria population involved in sludge hydrolysis and acidification at pH 10 in comparison to uncontrolled pH, as well as a decreased methanogenic archaea, which lead to a higher VFA production under this pH. Figure 7b shows the average percentage of every single VFA at alkaline conditions where it is observed that during periods B2 to B4, the predominant concentration shifted completely to acetic acid, which increased from 82.0 to 90.9%. This might be due to the preference of phosphoroclastic degradation pathway and these findings had been reported in some literatures (Agler et al. 2011; Dahiya et al. 2015; Garcia-Aguirre et al. 2017) showing values up to nearly 75% of acetic acid. Moreover, acetic acid percentage was even higher than the one observed during the batch assays, suggesting an adaptation of microbial community under this new environment. This fermentation broth would be especially interesting for biological heterotrophic denitrification, where acetic acid is preferred, followed by butyric and propionic acid (Elefsiniotis and Wareham 2007).

After day 57, the fermenter working at pH 10 was stopped for operation since it was clearly observed that these conditions highly favored acetic acid domination in the fermentation broth. Therefore, only the fermenter operating at pH 6 was kept in order to assess the evolution of total VFA afterwards. This fermenter at pH 6 was under observation to further investigate the relation between collection period and total VFA production. The VFA production started to increase after the 60th day, varying between 4.39 and 11.5 g VFA L^−1^, which was related to an increasing VS concentration in the feedstock (from 4.1% in period B4 to 6.1% in period B6 and afterwards nearly maintained in this value). These results also proved that an increase in OLR maintaining the HRT lead to higher VFA production, but in this study did not highly affect the VFA distribution, as observed in Fig. 7a. Between periods B3 and B8, acetic acid was the dominant product among VFA (49.6–60.3%), followed by caproic acid (27.0–28.3%) and butyric acid (10.9–12.6%). These results are consistent with those obtained by Venkateswar Reddy and Venkata Mohan (2012) when working with fresh unfermented FW and residual fermented FW. The concentrations of propionic and valeric acids were minority in periods between B3 and B8, and low valeric acid production could reflect the FW had small amount of protein (Shen et al. 2017). Moreover, during these periods, the ratio COD_VFA_/COD_s_ ranged from 14.6 to 29.5% which are similar values than the obtained when working with OFMSW.

In summary, higher VFA production was observed during semi-continuous acidogenic fermentation of FW at pH 9.5–10 when compared to pH 6 and the acetic acid concentration represented up to 91% of the total VFA produced. The implementation of pH near 6 in the acidogenic fermentation of FW generated an effluent with a stable composition distribution of acetic and butyric acids (up to 60.3% and 12.9%, respectively) during more than 70 days of operation (periods B4 to B8).

## Conclusions

The source-sorted OFMSW collected from a full-scale MBT plant had a VS content of 4.18–5.15% and a VFA concentration in the range of 8.6–10.1 g L^−1^. During mesophilic acidogenic fermentation of this substrate working at an HRT of 3.5 days at a pH near 6 without external chemical addition, the VFA concentration was increased by 13.9–16.9% and had a composition of individual VFA similar to that of influent (C_2_ 35–41%, C_3_ 18–22%, C_4_ 17–21%, and C_5_ 9–12%). Therefore, this effluent had a proportion (C_2_ + C_4_)/(C_3_ + C_5_) ≈ 1.7–2.0 and COD_VFA_/sCOD around 23%. A dilution assay revealed that there was no inhibition due to VFA in the fermenter working at acidic conditions.

Although a higher solubilization of COD was obtained under alkaline conditions (18.2–23.2% higher than that in acidic pH), no improvement in the VFA production was detected when using OFMSW, which could be caused by a high NH_4_^+^–N concentration (≥ 2.0 g N L^−1^) leading to free ammonia inhibition. This unusual high alkalinity and ammonium content in the collected OFMSW was related to the fact that OFMSW was mixed in the MBT plant with supernatant from anaerobic digestion of this biowaste.

Anaerobic batch tests using FW from a university canteen revealed that, in the acidic range of pH tested (4–6), improved VFA production was obtained at pH 6. However, higher VFA production was obtained at pH 9, with a higher content in acetic acid, which was confirmed when working with semi-continuous fermenters treating FW.

In the semi-continuous acidogenic fermentation of FW, the mesophilic digesters experienced a 30-day start-up period before a more stable VFA production was obtained. Under alkaline conditions, ammonia inhibition was avoided due to relatively low NH_4_^+^–N concentration (≤ 0.7 g N L^−1^) and 14–16% higher COD solubilization was observed with respect to acidic conditions, which lead to a higher VFA production characterized by a high content of acetic acid, up to 91%. When working under acidic conditions, an effluent with a VFA concentration up to 11.5 g VFA L^−1^ (FW with a VS content of 5.5% *w*/*w*) and a stable distribution of C_2_ and C_4_ acids (up to 60.3% and 12.9%, respectively) but with very low quantities of C_3_ and C_5_ acids (lower than 1.8 and 2.7%, respectively) were obtained, with a ratio COD_VFA_/sCOD that could reach values around 29.5%.

## Electronic supplementary material

ESM 1 (DOCX 29 kb)
