# Supplementary material for: Volatile fatty acid production from mesophilic acidogenic fermentation of organic fraction of municipal solid waste and food waste under acidic and alkaline pH
Source: Environ Sci Pollut Res Int. 2019 May 20;26(35):35509–22. doi: 10.1007/s11356-019-05394-6 (PMC6923264; doi:10.1007/s11356-019-05394-6)
Supplement: Supplementary file 1 — (DOCX 29 kb) [file 11356_2019_5394_MOESM1_ESM.docx]

**Volatile fatty acids production from mesophilic acidogenic fermentation of organic fraction of municipal solid waste and food waste under acidic and alkaline pH**

Yen-Keong Cheah^1^, Carme Vidal-Antich^1^, Joan Dosta^1,*^, Joan Mata-Álvarez^1,2^

^1^Department of Chemical Engineering and Analytical Chemistry, University of Barcelona, Barcelona, Catalonia, 08028, Spain.

^2^Water Research Institute, University of Barcelona, Catalonia, 08001, Spain.

*Corresponding author: [jdosta@ub.edu](mailto:jdosta@ub.edu)

**Environmental Science and Pollution Research**

**SUPPLEMENTARY MATERIAL**

**Table SI -** VFA composition on COD basis in the semi-continuous fermenter effluent treating OFMSW at acidic pH (6) and alkaline pH (10) under mesophilic conditions

| **Parameters** | **Units** | **Mesophilic fermenter at acidic pH (6)** | | | **Mesophilic fermenter at alkaline pH (10)** | | |
| --- | --- | --- | --- | --- | --- | --- | --- |
|  |  | **Period A1** | **Period A2** | **Period A3** | **Period A1** | **Period A2** | **Period A3** |
| VFA | gCOD L^-1^ | 17.52 ± 0.85 | 14.88 ± 1.38 | 17.36 ± 1.05 | 17.04 ± 0.74 | 14.78 ± 1.54 | 16.83 ± 2.35 |
| Acetic acid | % | 25.95 ± 0.84 | 28.48 ± 1.51 | 27.19 ± 2.54 | 26.67 ± 0.96 | 28.85 ± 1.08 | 28.88 ± 2.06 |
| Propionic acid | % | 19.66 ± 0.46 | 20.58 ± 1.25 | 17.78 ± 0.38 | 19.68 ± 0.41 | 20.76 ± 1.03 | 17.89 ± 1.04 |
| Isobutyric acid | % | 3.49 ± 0.16 | 5.15 ± 0.90 | 3.13 ± 0.99 | 3.52 ± 0.36 | 3.96 ± 0.34 | 2.68 ± 1.20 |
| Butyric acid | % | 18.49 ± 0.88 | 14.61 ± 1.76 | 20.22 ± 3.19 | 19.26 ± 0.45 | 15.57 ± 2.35 | 22.29 ± 3.05 |
| Isovaleric acid | % | 3.14 ± 0.52 | 3.59 ± 0.19 | 3.52 ± 0.27 | 2.35 ± 0.28 | 2.93 ± 0.62 | 1.95 ± 0.32 |
| Valeric acid | % | 11.05 ± 0.40 | 9.29 ± 0.88 | 11.63 ± 1.27 | 11.26 ± 0.27 | 9.63 ± 0.82 | 11.76 ± 2.04 |
| Isocaproic acid | % | 1.98 ± 0.09 | 2.68 ± 0.49 | 1.71 ± 0.41 | 2.05 ± 0.06 | 2.54 ± 0.56 | 3.33 ± 3.10 |
| Caproic acid | % | 6.30 ± 0.78 | 5.66 ± 0.56 | 7.96 ± 1.54 | 5.48 ± 0.53 | 5.24 ± 0.38 | 6.78 ± 0.49 |
| Heptanoic acid | % | 9.94 ± 1.27 | 9.95 ± 1.55 | 6.86 ± 2.65 | 9.74 ± 0.90 | 10.54 ± 1.60 | 4.44 ± 1.74 |

**Table SII** VFA composition on COD basis in the semi-continuous fermenter effluent treating FW at acidic pH (6) under mesophilic conditions

| **Parameters** | **Units** | **Effluent at pH 6** | | | | | | | |
| --- | --- | --- | --- | --- | --- | --- | --- | --- | --- |
|  |  | **Period B1** | **Period B2** | **Period B3** | **Period B4** | **Period B5** | **Period B6** | **Period B7** | **Period B8** |
| VFA | g COD L^-1^ | 6.82 ± 1.85 | 20.12 ± 3.25 | 7.60 ± 2.64 | 6.81 ± 1.80 | 8.55 ± 1.24 | 12.22 ± 1.26 | 14.12 ± 0.75 | 14.72 ± 1.43 |
| Acetic acid | % | 51.60 ± 18.71 | 21.04 ± 2.30 | 34.52 ± 7.71 | 42.15 ± 4.86 | 34.15 ± 7.76 | 42.09 ± 3.74 | 44.32 ± 2.55 | 42.74 ± 3.41 |
| Propionic acid | % | 10.78 ± 5.90 | 7.88 ± 2.20 | 2.83 ± 0.68 | 1.18 ± 1.18 | 1.71 ± 0.31 | 1.25 ± 0.09 | 1.30 ± 0.12 | 1.00 ± 0.53 |
| Isobutyric acid | % | 3.79 ± 1.54 | 2.13 ± 0.20 | 1.89 ± 0.99 | 1.10 ± 0.19 | 0.93 ± 0.12 | 0.87 ± 0.07 | 0.91 ± 0.13 | 0.87 ± 0.13 |
| Butyric acid | % | 11.07 ± 4.81 | 33.79 ± 0.77 | 11.68 ± 1.49 | 12.34 ± 2.36 | 15.09 ± 2.12 | 13.12 ± 2.44 | 15.54 ± 1.89 | 12.72 ± 1.51 |
| Isovaleric acid | % | 4.03 ± 1.19 | 3.87 ± 0.45 | 3.23 ± 1.52 | 2.45 ± 0.71 | 2.22 ± 0.26 | 2.48 ± 0.41 | 2.64 ± 0.35 | 2.81 ± 0.40 |
| Valeric acid | % | 6.88 ± 3.03 | 8.52 ± 0.98 | 2.24 ± 1.12 | 1.26 ± 0.27 | 1.07 ± 0.15 | 0.73 ± 0.06 | 0.83 ± 0.05 | 0.73 ± 0.06 |
| Isocaproic acid | % | 3.04 ± 1.31 | 0.37 ± 0.50 | 1.27 ± 1.37 | 0.11 ± 0.10 | 0.14 ± 0.05 | 0.07 ± 0.02 | 0.07 ± 0.01 | 0.06 ± 0.01 |
| Caproic acid | % | 4.77 ± 1.58 | 18.53 ± 1.56 | 38.01 ± 3.52 | 37.34 ± 3.45 | 42.88 ± 6.61 | 38.04 ± 3.29 | 33.11 ± 2.75 | 37.60 ± 2.60 |
| Heptanoic acid | % | 4.03 ± 2.46 | 3.87 ± 0.98 | 4.33 ± 2.95 | 2.06 ± 0.33 | 1.82 ± 0.24 | 1.34 ± 0.13 | 1.29 ± 0.08 | 1.46 ± 0.18 |

**Table SIII** VFA composition on COD basis in the semi-continuous fermenter effluent treating FW at alkaline pH under mesophilic conditions

| **Parameters** | **Units** | **Effluent at pH 10 (with control)** | | | |
| --- | --- | --- | --- | --- | --- |
|  |  | **Period B1** | **Period B2** | **Period B3** | **Period B4** |
| VFA | g COD L^-1^ | 7.53 ± 1.96 | 12.06 ± 1.50 | 8.07 ± 2.00 | 7.19 ± 1.67 |
| Acetic acid | % | 43.61 ± 12.97 | 71.25 ± 2.32 | 72.61 ± 15.75 | 84.83 ± 1.51 |
| Propionic acid | % | 12.98 ± 4.00 | 3.43 ± 0.76 | 2.85 ± 0.70 | 2.60 ± 0.35 |
| Isobutyric acid | % | 3.53 ± 1.27 | 2.14 ± 0.22 | 3.00 ± 0.60 | 2.57 ± 0.26 |
| Butyric acid | % | 17.24 ± 5.06 | 10.06 ± 2.24 | 7.21 ± 3.89 | 3.53 ± 0.48 |
| Isovaleric acid | % | 4.09 ± 1.44 | 3.65 ± 0.77 | 5.53 ± 0.73 | 4.54 ± 0.21 |
| Valeric acid | % | 6.67 ± 3.21 | 2.49 ± 0.16 | 1.48 ± 1.53 | 0.28 ± 0.63 |
| Isocaproic acid | % | 2.87 ± 2.89 | 1.94 ± 0.26 | 1.30 ± 1.48 | 0.00 ± 0.00 |
| Caproic acid | % | 4.83 ± 1.80 | 3.05 ± 0.22 | 4.19 ± 8.57 | 0.90 ± 0.32 |
| Heptanoic acid | % | 4.18 ± 2.42 | 2.00 ± 0.20 | 1.82 ± 1.51 | 0.75 ± 0.01 |
